# Humanized *Saccharomyces cerevisiae* provides a facile and effective tool to identify damaging human variants that cause exosomopathies

**DOI:** 10.1093/g3journal/jkaf036

**Published:** 2025-02-21

**Authors:** Khondakar Sayef Ahammed, Milo B Fasken, Anita H Corbett, Ambro van Hoof

**Affiliations:** Department of Microbiology and Molecular Genetics and MD Anderson UTHealth Houston Graduate School of Biomedical Sciences, University of Texas Health Science Center at Houston, Houston, TX 77030, USA; Department of Biology, Emory College of Arts and Sciences, Emory University, Atlanta, GA 30322, USA; Department of Biology, Emory College of Arts and Sciences, Emory University, Atlanta, GA 30322, USA; Department of Microbiology and Molecular Genetics and MD Anderson UTHealth Houston Graduate School of Biomedical Sciences, University of Texas Health Science Center at Houston, Houston, TX 77030, USA

**Keywords:** RNA exosome, RNA processing, RNase, *Saccharomyces cerevisiae*

## Abstract

The RNA exosome is an evolutionarily conserved, multiprotein complex that is the major RNase in 3′ processing and degradation of a wide range of RNAs in eukaryotes. Single amino acid changes in RNA exosome subunits cause rare genetic diseases collectively called exosomopathies. However, distinguishing disease-causing variants from nonpathogenic ones remains challenging, and the mechanism by which these variants cause disease is largely unknown. Previous studies have employed a budding yeast model of RNA exosome-linked diseases that relies on mutating the orthologous yeast genes. Here, we develop a humanized yeast model of exosomopathies that allows us to unambiguously assess damaging effects of the exact patient variant in budding yeast. Individual replacement of the yeast subunits with corresponding mammalian orthologs identified 6 out of 9 noncatalytic core subunits of the budding yeast RNA exosome that can be replaced by a mammalian subunit, with 3 of the replacements supporting close to normal growth. Further analysis of the disease-associated variants utilizing the hybrid yeast/mammalian RNA exosome revealed functional defects caused by both previously characterized and uncharacterized variants of EXOSC2, EXOSC4, EXOSC7, and EXOSC9. Analysis of the protein levels of these variants indicates that a subset of the patient-derived variants causes reduced protein levels, while other variants are defective but are expressed as well as the reference allele, suggesting a more direct contribution of these residues to RNA exosome function. This humanized yeast model of exosomopathies provides a convenient and sensitive genetic tool to help distinguish damaging RNA exosome variants from benign variants. This disease model can be further exploited to uncover the underpinning mechanism of RNA exosome defects.

## Graphical Abstract

**Graphical Abstract:**
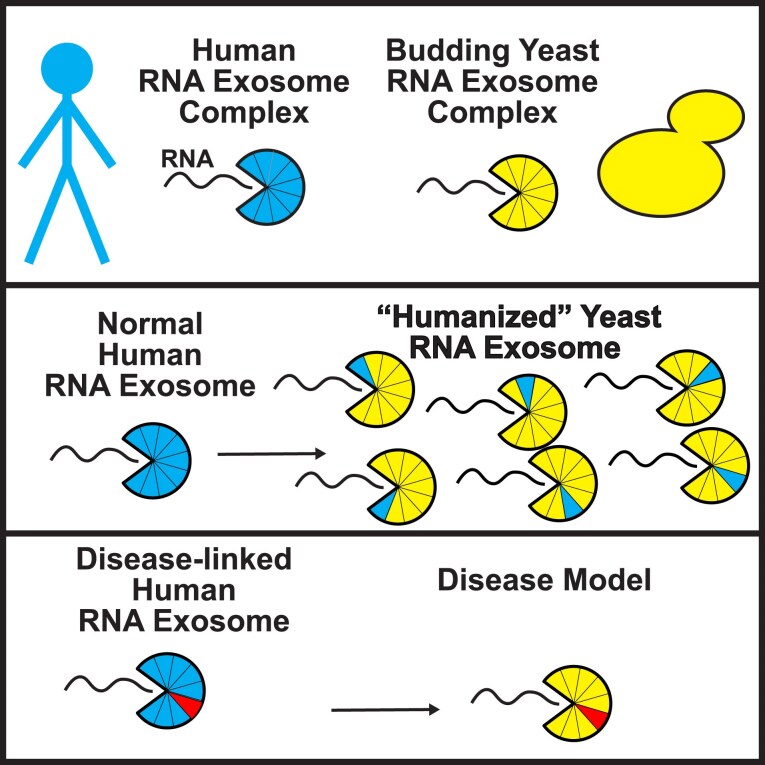

## Introduction

The RNA exosome complex is a major 3′–5′ exoribonuclease that plays a pivotal role in RNA processing and degradation in eukaryotes (Schneider and Tollervey 2013, Fig. 2b; Januszyk and Lima 2014). In the cytoplasm, the major function of the RNA exosome is to degrade incorrectly processed mRNAs (Schaeffer *et al.* 2011). The nuclear RNA exosome is involved in key steps in rRNA processing as well as the degradation of a wide range of noncoding RNAs (Mitchell *et al.* 1996; Schneider *et al.* 2012; Kilchert *et al.* 2016). Recent studies have highlighted the role of the RNA exosome complex in mammalian early development (Belair *et al.* 2019; Wu *et al.* 2019; Wu and Dean 2020, 2023; Laffleur *et al.* 2022; Petit *et al.* 2022; Yang *et al.* 2022; Srinivasan *et al.* 2024). However, we are only beginning to understand how specific RNA exosome substrates contribute to mammalian development.

**Fig. 1.**
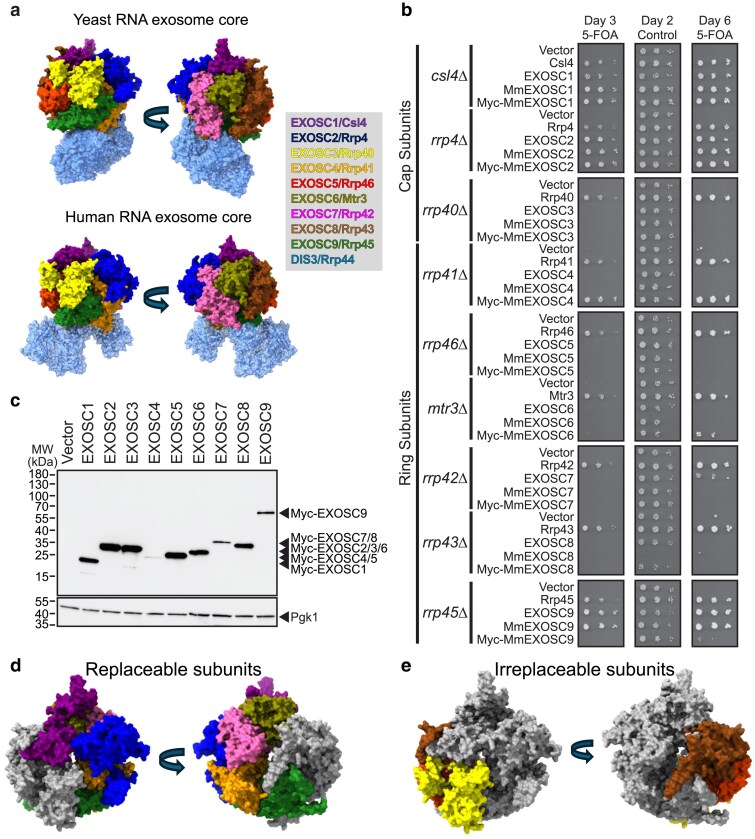
Selective yeast RNA exosome subunits can be replaced by human and mouse homologs. a) The budding yeast and human RNA exosome form similar complexes. The top panel shows a structure of the budding yeast RNA exosome Exo9 complex with Dis3/Rrp44 (PDB 6FSZ) (Schuller *et al.* 2018). The bottom panel shows the equivalent human structure (PDB 6D6R) (Weick *et al.* 2018). Orthologous subunits are shown in the same color as indicated. b) Functional complementation of human and mouse (Mm) core RNA exosome subunits (EXOSC1-9) in the corresponding budding yeast deletion mutants identifies the individually replaceable and irreplaceable subunits. Each human, mouse, or budding yeast RNA exosome subunit was expressed in a yeast strain containing a genomic deletion of the gene for the orthologous subunit and complemented by the wild-type yeast gene on a *URA3* plasmid. Serial dilution growth assays were performed on media containing 5-FOA, which selects for the yeast cells that have lost the *URA3* plasmid encoding the yeast subunit. Growth of the yeast strains was recorded on days 2, 3, and 6 as indicated. Growth on 5-FOA indicates that the human or mouse RNA exosome subunit can replace the essential yeast subunit. c) Steady-state protein level of EXOSC subunits does not explain the ability to complement. The steady-state protein level of indicated myc-tagged mouse RNA exosome subunits (EXOSC1-9) expressed in yeast was assessed by immunoblotting with an anti-myc antibody to compare the steady-state levels of the individual subunits. Pgk1 serves a loading control. d) and e) Structural representations and locations of replaceable and irreplaceable subunits. The replaceable subunits are colored in d and the irreplaceable are in gray. Colors correspond to those in a. e shows the complementary view with the irreplaceable subunits colored.

**Fig. 2.**
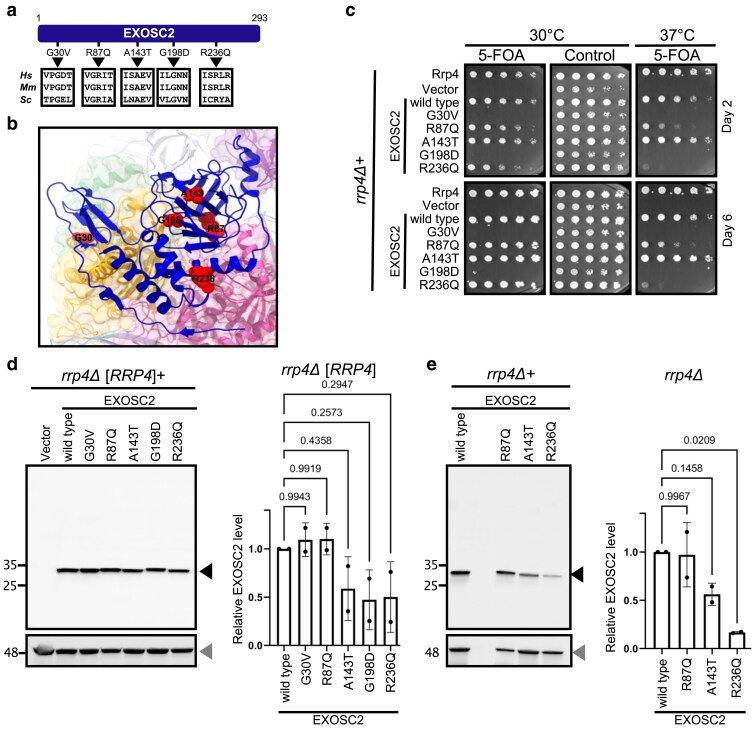
A humanized budding yeast model to assess functional defects in SHRF-associated EXOSC2 variants. a) Schematic representation of the analyzed SHRF-associated amino acid changes in human EXOSC2 (*Hs*). The corresponding amino acid residues in the mouse (*Mm*) and budding yeast (*Sc*) sequences are indicated with arrowheads. b) The locations of the SHRF-associated variant amino acid residues (depicted space-filled in red) in EXOSC2 (depicted as ribbons in blue) are shown in the RNA exosome structure (PDB 6D6R) (Weick *et al.* 2018). c) Effect of SHRF-associated EXOSC2 variants on the ability of EXOSC2 to complement *rrp4Δ*. The indicated human EXOSC2 variants were expressed in an *rrp4Δ* yeast strain and subjected to plasmid shuffle experiments on media containing 5-FOA or control media. The growth of individual strains was compared with those that express either control yeast Rrp4 or wild-type EXOSC2 at the indicated days and temperatures. Comparison of the steady-state protein levels of EXOSC2 variants expressed in the presence (d) and absence (e) of yeast Rrp4. The EXOSC2 protein (black arrowhead) level for indicated strains was quantitated, plotted following normalization to Pgk1 (loading control; gray arrowhead), and compared with wild-type EXOSC2 level. The average of 2 biological replicates is plotted (bar) as well as the data points corresponding to the individual replicates (circles). *P*-values were determined by one-way ANOVAs.

The structure of the RNA exosome core is very well conserved among eukaryotes, and many detailed studies have revealed the structure of a conserved core of 9 subunits, named Exo9 (Makino *et al.* 2013; Januszyk and Lima 2014; Kilchert *et al.* 2016; Schmid and Jensen 2019). Six of the core subunits are distant homologs of each other and form a barrel-shaped central ring. These subunits are termed EXOSC4 to EXOSC9 in humans and Rrp41, Rrp46, Mtr3, Rrp42, Rrp43, and Rrp45 in budding yeast (Fig. 1a). This hexameric ring is stabilized by 3 cap subunits, EXOSC1 to EXOSC3, or Rrp4, Rrp40, and Csl4. The Exo9 core associates with several 3′–5′ exoribonuclease subunits that provide the catalytic functions: DIS3, DIS3L, and EXOSC10 in humans and Rrp44/Dis3 and Rrp6 in budding yeast. In addition, the core RNA exosome interacts with several cytoplasmic- and nuclear-specific cofactors that facilitate RNA channeling through its central ring toward the DIS3/DIS3L/Rrp44 active site.

Null mutations in the genes that encode the Exo9 subunits are lethal in all eukaryotes where this has been tested (Fasken *et al.* 2020). While null mutations in humans presumably would not result in a live birth, a number of single amino acid variants in 7 of the 9 structural RNA exosome subunits have been clinically linked to a range of Mendelian diseases, collectively referred to as “exosomopathies” (Morton *et al.* 2018; Fasken *et al.* 2020). So far, variants of 4 subunits (EXOSC1, EXOSC3, EXOSC8, and EXOSC9) have been linked to a collection of neurodevelopmental diseases named pontocerebellar hypoplasia type 1 (PCH1) (Wan *et al.* 2012; Boczonadi *et al.* 2014; Burns *et al.* 2018; Somashekar *et al.* 2021; Zaki *et al.* 2024). EXOSC4 has been linked to a neurodevelopmental disorder with some mild brain atrophy reported (Fasken *et al.* 2024). EXOSC5 variants are associated with a disease named cerebellar ataxia, brain abnormalities, and cardiac conduction defects (CABAC) (Di Donato *et al.* 2016; Slavotinek *et al.* 2020). CABAC and PCH share significant similarities. While these disease all share abnormal brain development, a clearly distinct set of clinical features, short stature, hearing loss, retinitis pigmentosa, and distinctive facies (SHRF) is caused by EXOSC2 variants (Di Donato *et al.* 2016). It is unclear why EXOSC2 variants cause a different disease and why EXOSC6 and EXOSC7 disease-causing variants have not been identified (for a full discussion, see recent reviews by Morton *et al.* 2018 and Fasken *et al.* 2020). While exome sequencing of patients has facilitated the identification of RNA exosome variants, distinguishing disease-causing variants from benign variants remains challenging. Defining how the identified amino acid substitutions in the patients affect RNA exosome structural integrity and function is an ongoing research focus. Both of these challenges are in part due to limited genetic model systems to identify the damaging effects of, and to functionally analyze, exosome subunit variants.

To address these challenges, we and others have previously implemented the “budding yeast model” of RNA exosome-linked diseases by introducing patient variants in orthologous yeast RNA exosome subunits (Fasken *et al.* 2017, 2024; Gillespie *et al.* 2017; Slavotinek *et al.* 2020; Sterrett *et al.* 2021, 2023). For example, EXOSC2-G198D causes disease and has been modeled with an *rrp4-G226D* mutation that causes slow growth (Sterrett *et al.* 2021). These yeast models have been valuable in detecting the damaging effects of some RNA exosome variants and have provided important insights into molecular mechanisms of RNA exosome defects. However, a considerable number of orthologous mutations in the budding yeast RNA exosome subunits show little or no effect. In other cases, it is difficult to unambiguously identify orthologous mutations due to differences in amino acid sequences between budding yeast and human RNA exosome subunits. In a different approach, we have previously shown that the disease-linked EXOSC1 variants EXOSC1-S35L and EXOSC1-R183W are damaging by expressing the human variants in yeast (Damseh *et al.* 2023), instead of creating the orthologous yeast mutations.

Here, we developed a “humanized yeast model” for other Exo9 subunits in order to analyze the damaging effects of disease-linked RNA exosome variants. First, we systematically tested which individual yeast RNA exosome subunits could be replaced with mammalian (human and mouse) orthologs. As missense mutations in several replaceable subunits are linked to a variety of genetic diseases, we have further utilized the humanized budding yeast as a platform for identifying functional defects of the RNA exosome caused by patient variants. In this study, we assayed RNA exosome defects caused by previously known as well as previously uncharacterized patient-derived variants in EXOSC2, EXOSC4, EXOSC7, and EXOSC9. Some variants that show no or minor defects in the yeast model under standard growth conditions show a greater effect in this humanized yeast model. Furthermore, we can now analyze the effects of amino acid changes in residues that were difficult to align between yeast and humans. Thus, the “humanized yeast model” is a sensitive and convenient tool to help determine the effects of disease-associated variants on RNA exosome function.

## Materials and methods

### Plasmids and *Saccharomyces cerevisiae* strains

The plasmids used in this study are listed in Supplementary Table 1. The human EXOSC1 complementing plasmid has previously been described (Schaeffer *et al.* 2009). The codon-optimized ORFs encoding human EXOSC2-9 were obtained from GenScript and subcloned into the p415TEF plasmid using XhoI and BamHI restriction sites (Mumberg *et al.* 1995). The untagged and N-terminal myc-tagged mouse RNA exosome ORFs (MmEXOSC1-9) were amplified from mouse N2a cell cDNA and cloned into pRS315 (Sikorski and Hieter 1989) containing a *TEF1* promoter and a *CYC1* terminator using NEBuilder HiFi DNA Assembly Master Mix (New England Biolabs). The *S. cerevisiae* RNA exosome ORFs with native promoters and terminators were cloned into pRS415 or pRS315 (for expression of the yeast subunits in deletion strains) and pRS416 or pRS316 (for rescue plasmid to construct the yeast deletion strains). The pRS31x and pRS41x plasmids used in parallel experiments by the AHC and AvH labs differ by the orientation of the multiple cloning site and gave equivalent results (Sikorski and Hieter 1989). The plasmids encoding the disease-linked variants of EXOSC2, MmEXOSC4, EXOSC7, and EXOSC9 were created using the QuikChange Lightning Site-Directed Mutagenesis Kit following the manufacturer's instructions. The oligonucleotide primers used for amplifying the genes and for mutagenesis are listed in Supplementary Table 2. The sequences of the human and mouse EXOSC plasmids were verified by whole plasmid nanopore sequencing (Plasmidsaurus) and/or Sanger sequencing (McLab). The mutant plasmids were confirmed by Sanger sequencing (Genewiz).

The yAv yeast deletion strains of RNA exosome subunit genes (listed in Supplementary Table 3) were created from the heterozygous mutant library (Giaever *et al.* 2002). In short, the rescue plasmids encoding the wild-type yeast RNA exosome subunits containing the *URA3* counter-selectable marker were transformed into the corresponding yeast diploid heterozygous mutants and subjected to sporulation. Haploid neomycin-resistant and 5-FOA-sensitive progeny were identified and used in further experiments. In parallel, ACY yeast strains (listed in Supplementary Table 3) were created by transforming BY4741 with a *RRP/URA3* plasmid and subsequently disrupting the endogenous RRP gene with a KanMX marker. Both sets of strains gave equivalent results.

### Structural analysis and pathogenicity prediction

The structural analysis of the RNA exosome subunits and variants described in this study was performed with UCSF ChimeraX using the cryoEM structure of the human RNA exosome complex (PDB: 6D6R) and yeast RNA exosome complex (PDB: 6FSZ) (Schuller *et al.* 2018; Weick *et al.* 2018). Pathogenicity prediction of the disease-linked variants was performed using the AlphaMissense database and PolyPhen-2 (Adzhubei *et al.* 2013; Cheng *et al.* 2023). Prediction of the protein stability of the RNA exosome variants was performed using DDMut and mCSM webservers (Pires *et al.* 2014; Zhou *et al.* 2023).

### Functional complementation and growth assays

To identify the replaceable and irreplaceable subunits of the yeast RNA exosome, the plasmids encoding human, mouse, or yeast subunits were transformed into the corresponding yeast subunit deletion mutants (already containing a *RRP*/*URA3* plasmid). The transformants were selected in SC media lacking leucine and uracil (SC-Leu-Ura, Sunrise Science). A series of plasmid shuffle assays were carried out for each subunit by serially diluting the strains containing empty vector control, yeast, human, or mouse RNA exosome components. The strains were spotted on 5-FOA-containing media to select for cells that had lost the *URA3* plasmid encoding the wild-type yeast subunit. Growth under these conditions indicates that the human or mouse RNA exosome subunit can substitute for the essential yeast subunit. Growth of the strains was recorded on days 3 and 6 at 30°C. Strains were also spotted on YPD or SC-Leu for control.

To test the complementation of the RNA exosome variants, the corresponding deletion strains were transformed with the wild-type and mutant EXOSC plasmids. The growth of the serially diluted and spotted yeast was recorded on days 2 and 6 at 30 and 37°C.

### Immunoblotting of the RNA exosome subunits

The yeast strains that express wild-type or variant human or mouse exosome subunits were grown to an OD_600_ of ∼0.6–0.8 at 30°C. The yeast strains that express human EXOSC subunits were resuspended in IP50 (50 mM Tris–HCl pH 7.5, 50 mM NaCl 2 mM MgCl_2_, 0.1% Triton X-100, 0.5 mM β-mercaptoethanol, and 0.1 mM PMSF, with complete EDTA-free protease inhibitors [Roche]). The yeast strains that express mouse EXOSC subunits were resuspended in RIPA-2 (50 mM Tris–HCl pH 8, 150 mM NaCl, 0.5% sodium deoxycholate, 1% NP40, 0.1% SDS) supplemented with protease inhibitors (1 mM PMSF, Pierce Protease Inhibitors, ThermoFisher Scientific). Cells were lysed by vortexing with acid-washed glass beads, and lysates were collected upon centrifugation. The protein samples were subjected to SDS–PAGE and western blot. The human and mouse RNA exosome subunits were probed using rabbit polyclonal primary antibodies obtained from Proteintech and Bethyl: anti-EXOSC2 (cat. no. 14805-1-AP), anti-EXOSC4 (cat. nos. 15937-1-AP and A303-774A), anti-EXOSC7 (cat. no. 25292-1-AP), and anti-EXOSC9 (cat. no. 24470-1-AP). The western blots of the yeast strains that express myc-tagged mouse RNA exosome subunits were probed using monoclonal antibody 9B11 (cat. no. 2276S; Cell Signaling Technology). Secondary goat antirabbit (Biorad, cat. no. 1706515) or goat antimouse (Biorad, cat. no. 1706516) antibodies and WesternBright ECL-spray (VWR, cat. no. K-12049) were used to generate a signal that was detected on a Biorad ChemiDoc MP Imaging System and quantitated using Image Lab version 6.1. Band intensities were normalized to loading control Pgk1 (detected with anti-Pgk1 antibody, cat. no. 459250; Invitrogen). One-way ANOVAs of normalized protein levels were performed using GraphPad Prism.

## Results

### Selective budding yeast RNA exosome subunits can be replaced by human and mouse orthologs

We sought to test whether the human and mouse orthologs of RNA exosome subunits (EXOSC1-9) can individually replace the orthologous budding yeast RNA exosome subunit. We focused on the replaceability of the noncatalytic essential subunits of the yeast Exo9 RNA exosome core complex, i.e. the 3 cap subunits (Csl4, Rrp4, and Rrp40) and the 6 barrel subunits (Rrp41, Rrp46, Mtr3, Rrp42, Rrp43, and Rrp45). We focused on Exo9, because variants of the catalytic subunits that cause Mendelian disease had not yet been described when we initiated these studies. We had previously generated plasmids with mouse EXOSC1-9 ORFs for experiments in N2a cells that could readily be subcloned into yeast plasmids. For the human subunits, we did not have similar plasmids and decided to use synthetic ORFs that were codon optimized for budding yeast expression. Plasmids for both the human (EXOSC1-9) and mouse RNA exosome subunits (MmEXOSC1-9) were introduced into the corresponding yeast deletion strain. These yeast strains each have the endogenous gene encoding an RNA exosome subunit deleted from the chromosome and replaced with the respective budding yeast gene on a *URA3* counter-selectable plasmid. A series of plasmid shuffle assays were performed to replace one of the yeast RNA exosome subunits encoded on the *URA3* plasmid with the human or mouse counterpart.

We observed that only a subset of the RNA exosome subunits can be individually replaced by human or mouse counterparts (Fig. 1). For 3 of the 9 subunits (Csl4, Rrp4, and Rrp45), both the human and mouse orthologs supported growth that was similar to wild type. For 3 other subunits (Rrp40, Rrp43, and Rrp46), neither the human nor the mouse ortholog could replace them. For the remaining 3 subunits (Rrp42, Rrp42, and Mtr3), only one of the mammalian orthologs could complement and supported growth that was markedly slower than wild type. Among the 3 cap subunits, the human and mouse EXOSC1 or EXOSC2 can functionally complement the loss of yeast Csl4 or Rrp4 (Fig. 1b). However, the other cap subunit, Rrp40, appears irreplaceable by the homologous EXOSC3 protein. Among the 6 ring subunits, 4 can be replaced: Rrp41, Rrp42, Rrp45, and Mtr3. As shown in Fig. 1b, human EXOSC7 could weakly complement *rrp42Δ*, but the mouse counterpart could not. We also observed complementation of *rrp41Δ* and *mtr3Δ* with the N-terminally myc-tagged mouse myc-EXOSC4 and myc-EXOSC6 plasmids, respectively, while surprisingly, the untagged human or mouse genes did not complement. This result may be partially attributable to steady-state protein levels because the tagged mouse EXOSC4 protein is present at a 6-fold higher level than the untagged mouse EXOSC4 (Supplementary Fig. 1). Although the human EXOSC4 plasmid failed to complement *rrp41*Δ, this plasmid can complement the depletion of yeast Rrp41 in a strain where Rrp41 is expressed from a GAL promoter and then depleted by shifting the cells to glucose (Brouwer *et al.* 2001) (Supplementary Fig. 1). This result suggests that, perhaps, human EXOSC4 can substitute for some of the functions of Rrp41, but not for all of the functions. Taken together, 6 out of the 9 noncatalytic subunits of yeast RNA exosome are replaceable to some extent by their human or mouse counterpart.

One reason we used epitope-tagged mouse ORFs was to allow for comparison of steady-state protein levels of different RNA exosome subunits. We speculated that high steady-state protein levels of the RNA exosome subunits in the yeast cell might correlate with their ability to complement, while low levels might explain failure to complement. Because some of the MmEXOSC subunits failed to complement, we could only compare expression levels of all 9 subunits in yeast strains that also express the corresponding yeast subunit. We were able to detect each of the myc-tagged mouse RNA exosome subunits, although with some variation in protein level (Fig. 1c). Notably, the steady-state protein level did not show an obvious correlation with the ability to complement. Myc-MmEXOSC5 was among the most highly expressed subunits, but failed to complement. On the other hand, Myc-MmEXOSC4 was one of the least expressed subunits and complemented. Thus, the complementation outcome of the mouse RNA exosome subunits does not correlate with the steady-state protein level of the subunits when expressed in yeast cells.

We also compared the sequence similarity between the human and yeast subunits and observed that the replaceable subunits tend to be slightly less diverged in sequence than the irreplaceable ones. Specifically, the average identity between human and yeast for the 3 subunits that can be replaced by both human and mouse is 38%. The average similarity is 33% for the 6 subunits that can be replaced by at least one of the human or mouse plasmids and only 28% for the irreplaceable subunits (Supplementary Fig. 2). This analysis suggests that the more rapidly evolving subunits may be too diverged to complement. However, this correlation was not completely predictive because the irreplaceable Rrp40 is more similar to its human ortholog, EXOSC3, than the replaceable Rrp42 (32 vs 23%).

We observed that the RNA exosome appears to have distinct replaceable and irreplaceable sides (Fig. 1d and e). The replaceable subunits are depicted in color in Fig. 1d, whereas the irreplaceable ones are shown in gray. Conversely, in Fig. 1e, the replaceable ones are shown in gray and the irreplaceable ones are depicted in color. The replaceable side of the RNA exosome is composed of 4 ring subunits and 2 cap subunits (EXOSC1/Csl4, EXOSC2/Rrp4, EXOSC4/Rrp41, EXOSC6/Mtr3, EXOSC7/Rrp42, and EXOSC9/Rrp45). We did not observe a difference in localization within the RNA exosome of the 3 subunits that could be robustly replaced by both the human and mouse orthologs vs the 3 that could be replaced less well (Supplementary Fig. 3). Additionally, we found that the replaceable subunits have more extensive contacts with the catalytic subunit DIS3/Rrp44 than those that are irreplaceable. These data indicate that despite extensive sequence and structural similarities of the human and yeast subunits, amino acid level differences may play an important role in the functional complementation of the subunits. This might be due to disruption of the local protein–protein interactions between the human and yeast subunits, ultimately destabilizing the complex for the irreplaceable subunits.

### Humanized yeast allows assessment of functional defects in SHRF-associated RNA exosome variants

We employed the humanized yeast model for functional analysis of a number of disease-linked variants in RNA exosome subunits. We first analyzed 2 SHRF-associated RNA exosome variants, EXOSC2-G30V and EXOSC2-G198D (OMIM: 602238), that cause defects in RNA exosome function (Fig. 2a and b) (Di Donato *et al.* 2016; Yang *et al.* 2020). Previous functional analysis using the orthologous variants in the corresponding yeast subunit Rrp4 revealed that *rrp4-G58V* (corresponding to EXOSC2-G30V) is lethal and *rrp4-G226D* (corresponding to EXOSC2-G198D) shows a growth defect (Sterrett *et al.* 2021). To test whether expressing these disease-associated EXOSC2 variants in *rrp4Δ* yeast recapitulates results with disease-linked variants modeled in Rrp4, we expressed the EXOSC2-G30V and EXOSC2-G198D variants in an *rrp4Δ* yeast strain and compared the growth with the strain expressing either yeast Rrp4 or wild-type EXOSC2 in a plasmid shuffle assay. Both variants were unable to replace the essential Rrp4 subunit at 30 or 37°C when expressed as the sole copy, while wild-type EXOSC2 complemented the loss of Rrp4 (Fig. 2c). Thus, the expression of the human variants in a yeast deletion produced a similar defect for EXOSC2-G30V and a more severe defect for the EXOSC2-G198D variant compared with the previously published orthologous yeast models of these missense variants (Sterrett *et al.* 2021).

Previous publications have shown that the stability of the EXOSC2-G198D variant is affected in HEK293T cells, and the steady-state level of this variant is affected in SHRF patient B-lymphoblast cells and in the orthologous yeast models (Di Donato *et al.* 2016; Yang *et al.* 2020). In contrast, the EXOSC2-G30V was unaffected in these experiments. In line with previous observations, we found that the EXOSC2-G198D variant, but not the EXOSC2-G30V, shows reduced steady-state protein levels in yeast as compared to control EXOSC2 (Fig. 2d and e).

In addition to the previously characterized variants, we selected 3 uncharacterized EXOSC2 variants identified in patients with inborn genetic diseases to analyze (Fig. 2a and b). Specifically, an individual with clinical symptoms of SHRF was reported as compound heterozygous for EXOSC2 variants with a maternal missense mutation (EXOSC2-A143T) and a paternal splice donor variant (Reeves *et al.* 2022). Another report identified a compound heterozygous SHRF patient with 2 missense mutations (EXOSC2-R87Q and EXOSC2-R236Q) (Al-Kasbi *et al.* 2022). We explored the available databases used to predict the pathogenicity of genetic variants. Based on the AlphaMissense pathogenicity scores, EXOSC2-A143T and EXOSC2-R236Q are predicted to be “likely pathogenic,” whereas the EXOSC2-R87Q variant is categorized as “ambiguous” (Supplementary Table 4). None of these 3 variants were previously tested in any functional assays.

We expressed these uncharacterized human EXOSC2 variants in *rrp4Δ* cells and subjected them to a plasmid shuffle assay. Cells expressing the EXOSC2-A143T variant exhibit normal growth at all tested temperatures (30 and 37°C), similar to the strain expressing control Rrp4 or wild-type EXOSC2 (Fig. 2c). In contrast, the EXOSC2-R87Q and EXOSC2-R236Q variants confer variable degrees of growth defects. The EXOSC2-R87Q variant causes minimal growth defects at 30°C, but we observed a pronounced growth defect at 37°C. The EXOSC2-R236Q variant shows a growth defect at 30°C and does not support growth at 37°C. These results indicate that the EXOSC2-R87Q and EXOSC2-R236Q variants impair RNA exosome function in this humanized yeast model. Notably, the observed growth defects in the EXOSC2-R87Q and EXOSC2-R236Q variants are less pronounced compared with EXOSC2-G30V or EXOSC2-G198D, suggesting a partial loss of function in these variants.

We examined the protein levels of the 3 previously uncharacterized EXOSC2 variants (Fig. 2d and e). When expressed as the sole copy, the EXOSC2-A143T protein level was decreased slightly (∼2-fold) relative to control EXOSC2, while the EXOSC2-R236Q protein was more severely reduced relative to control EXOSC2. This result suggests that both of these variants are damaging and likely destabilize EXOSC2. For the viable EXOSC2-A143T variant, the protein level reduction appears sufficient to support normal yeast growth. Interestingly, the EXOSC2-R87Q variant is expressed at levels similar to wild-type EXOSC2, yet this variant causes a growth defect. This result suggests that EXOSC2-R87 might have a function other than conferring structural stability. In support of our experimental observations, the protein stability prediction tools DDMut and mCSM suggest that the EXOSC2 variants of interest are “destabilizing” with a varied range of predicted stability change scores (Supplementary Table 4). Overall, the humanized budding yeast model provides evidence that all 5 SHRF-linked missense variants are damaging and result in reduced growth and/or protein level.

We analyzed the position of the affected amino acid residues in the EXOSC2 protein using the published structure of the human RNA exosome (PDB:6D6R; Weick *et al.* 2018; Fig. 2b). EXOSC2 A143 (located in a β-strand) and R236 (located in an α-helix) residues both contact neighboring residues of EXOSC2. EXOSC2-R87 participates in intersubunit hydrogen bonding with a glutamate residue in the N-terminal domain of EXOSC7. Thus, the amino acid changes in the EXOSC2 variants may cause defects in RNA exosome function either by impairing the stability of EXOSC2 or by disrupting interactions with neighboring RNA exosome components.

### An EXOSC4 variant linked to neurodevelopmental defects impairs RNA exosome function

**Fig. 3.**
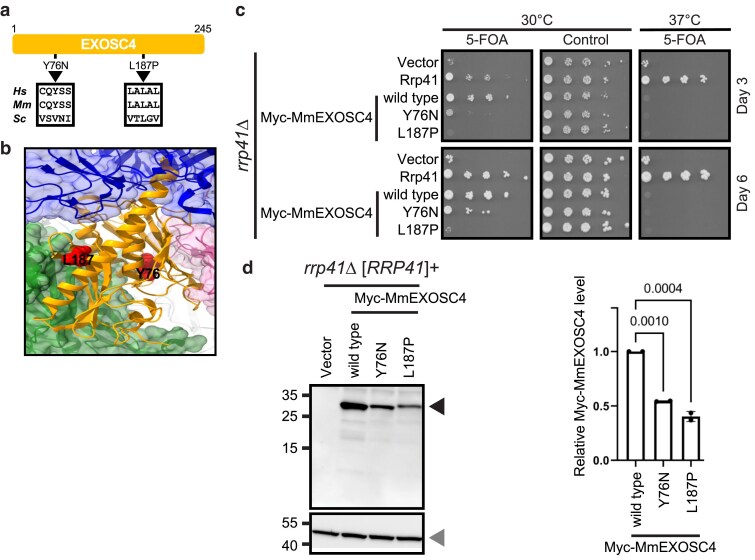
Functional defects in the RNA exosome associated with EXOSC4 variants are distinguishable in budding yeast. a) Schematic representation and corresponding amino acid substitutions of a previously reported EXOSC4 variant (EXOSC4-L187P) linked to neurodevelopmental defects and a novel variant of uncertain significance (EXOSC4-Y76N). The corresponding residues are also shown for the mouse (*Mm*) and budding yeast (*Sc*) orthologs. b) The location of the variant amino acid residues (depicted space-filled in red) in EXOSC4 (depicted as ribbons in orange) is indicated in the RNA exosome structure (PDB 6D6R). c) Effect of mouse myc-MmEXOSC4 variants on their ability to complement *rrp41Δ*. The myc-MmEXOSC4 variants were expressed in an *rrp41Δ* yeast strain and subjected to plasmid shuffle experiments on media containing 5-FOA or control media. The growth of individual strains was compared with those that express control Rrp41 or wild-type myc-tagged MmEXOSC4 at the indicated days and temperatures. d) The steady-state protein level of each myc-MmEXOSC4 variants was measured when expressed in the presence of yeast Rrp41. The normalized protein levels for the myc-tagged MmEXOSC4 variants (black arrowhead) were plotted and compared with the level of wild-type myc-tagged MmEXOSC4 following normalization to Pgk1 (loading control; gray arrowhead). The average of 2 biological replicates is plotted (bar) as well as the data points corresponding to the individual replicates (circles). *P*-values were determined by one-way ANOVAs.

An EXOSC4 variant has recently been added to the list of RNA exosome subunits linked to inborn neurodevelopmental disorders (Fasken *et al.* 2024). Two siblings with neurodevelopmental defects were reported to have a single amino acid change that replaces leucine 187 with proline in EXOSC4 (EXOSC4-L187P) (Fig. 3a and b). Functional analysis of the corresponding *rrp41-L187P* variant in the orthologous budding yeast subunit showed a decrease in the steady-state level of the protein and functional defects in the RNA exosome. We included another novel EXOSC4 variant in our study that replaces tyrosine 76 with asparagine (EXOSC4-Y76N). This variant was identified in 2 patients with microcephaly, but the symptoms could not be specifically attributed to the EXOSC4 variant because the patients also had a pathogenic variant in another protein, PAXBP1, that appears to explain the disease (Alharby *et al.* 2017). Both patient-derived missense EXOSC4 variants are categorized as “likely pathogenic” by the AlphaMissense pathogenicity prediction tool (Supplementary Table 4).

As human EXOSC4 cannot replace yeast Rrp41, we introduced orthologous amino acid changes in the mouse myc-tagged EXOSC4 and expressed the variants (MmEXOSC4-Y76N and MmEXOSC4-L187P) in the *rrp41Δ* yeast strain. Functional analysis showed that the myc-tagged MmEXOSC4-Y76N variant weakly complements the *rrp41Δ* mutation (Fig. 3c). In contrast, myc-tagged MmEXOSC4-L187P variant does not complement *rrp41Δ*. Thus, both the EXOSC4-Y76N and EXOSC4-L187P variants appear damaging.

The protein stability prediction tools (DDMut and mCSM webservers) suggest that both these EXOSC4 variants are “destabilizing.” Thus, we examined the steady-state protein levels of MmEXOSC4-Y76N and MmEXOSC4-L187P when expressed in the presence of Rrp41. Compared with the wild-type MmEXOSC4, a significant but moderate reduction to ∼54 and ∼40% is observed for MmEXOSC4-Y76N and MmEXOSC4-L187P, respectively (Fig. 3d). These data collectively indicate the damaging effect of the EXOSC4 variants on RNA exosome function. However, such a moderate reduction in protein levels suggests that perhaps both reduction in protein stability and disruption of protein–protein interaction with the neighboring subunits may contribute to the damaging effects of the variants.

### Identification of a damaging missense variant of EXOSC7

**Fig. 4.**
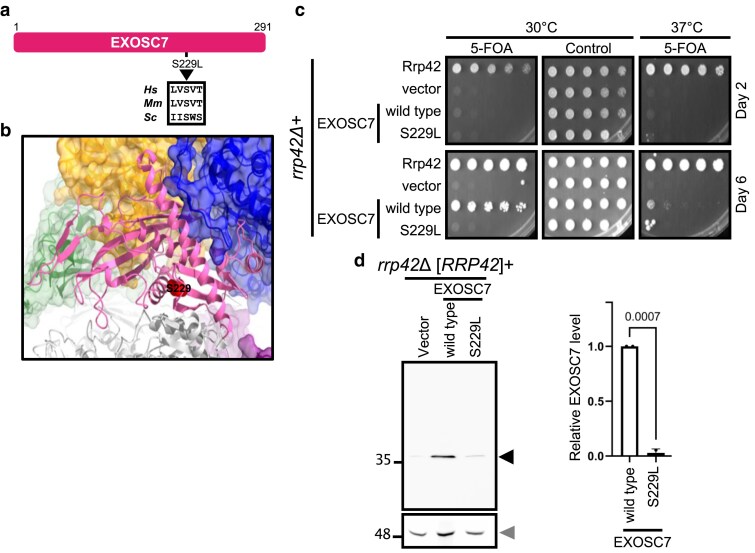
An EXOSC7 variant of uncertain significance is damaging in a humanized yeast model. a) Schematic representation and corresponding amino acid substitutions of a novel EXOSC7 variant of uncertain significance (EXOSC7-S229L). The corresponding residue is also shown for mouse (*Mm*) and budding yeast (*Sc*). b) The location of the variant amino acid residue (depicted space-filled in red) in EXOSC7 (depicted as ribbons in pink) is indicated in the RNA exosome structure (PDB 6D6R) (Weick *et al.* 2018). c) Effect of the EXOSC7 variant on ability of EXOSC7 to complement *rrp42Δ*. The EXOSC7-S229L variant was expressed in an *rrp42Δ* yeast strain and subjected to plasmid shuffle experiments on media containing 5-FOA or control media. The growth of the EXOSC7-S229L strain was compared to the strain expressing control Rrp42 or wild-type EXOSC7 at the indicated days and temperatures. d) The steady-state protein level of the EXOSC7-S229L variant was measured when expressed in the presence of yeast Rrp42. The normalized protein level for the EXOSC7 variant (black arrowhead) was plotted and compared with the level of wild-type EXOSC7 following normalization to Pgk1 (loading control; gray arrowhead). The average of 2 biological replicates is plotted (bar) as well as the data points corresponding to the individual replicates (circles). *P*-values were determined by one-way ANOVAs.

No association of EXOSC7 variants with disease symptoms has been published, but through the GeneMatcher service (Sobreira *et al.* 2015), we were notified by 3 different groups that they identified an EXOSC7-S229L variant in patients (Fig. 4a and b). These patients presented with diverse symptoms. One patient was described as having isolated diaphragmatic hernia, 1 patient as showing PCH phenotypes, and 1 patient as having congenital ataxia. Whether this variant is pathogenic and responsible for any of these symptoms was not clear. The AlphaMissense pathogenicity prediction tool was not helpful because it predicts this variant as “ambiguous” (Supplementary Table 4). We therefore tested whether the variant causes any defects in RNA exosome function. To achieve this goal, we expressed the wild-type human EXOSC7 and the EXOSC7-S229L variant in the *rrp42Δ* yeast strain and performed a plasmid shuffle assay (Fig. 4c). Whereas the wild-type EXOSC7 supports slow growth, the EXOSC7-S229L variant was unable to support growth when expressed as the sole EXOSC7 copy. Further investigation of protein levels indicates that the steady-state protein level of EXOSC7-S229L variant is significantly reduced compared with wild-type EXOSC7 (Fig. 4d). Although this variant has not been definitively linked to human disease, these results suggest it is damaging and indicate that damaging EXOSC7 variants can be identified using our humanized yeast model.

### PCH-linked EXOSC9 variants are defective in RNA exosome function

**Fig. 5.**
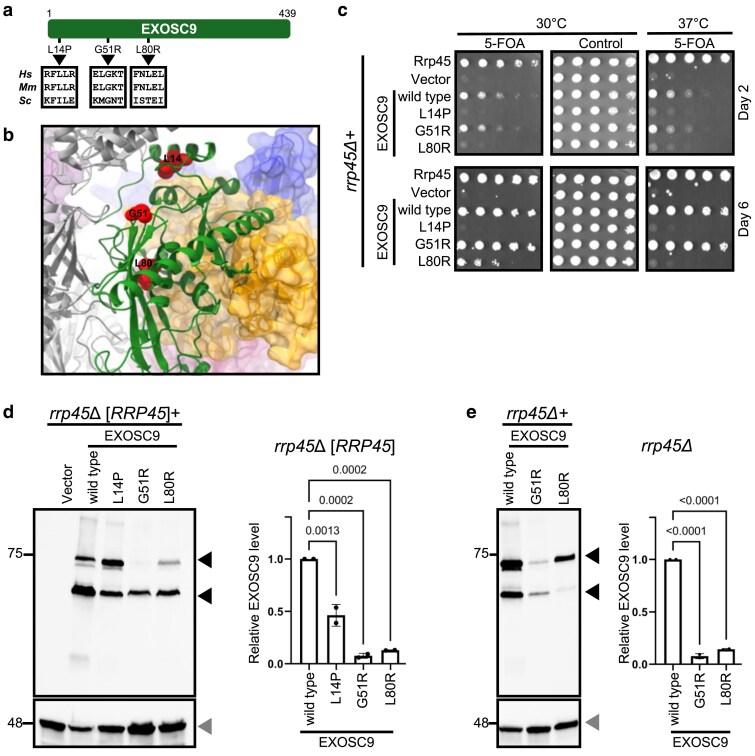
PCH-linked EXOSC9 variants are defective in a humanized yeast model. a) Schematic representation of PCH-associated EXOSC9 variants and corresponding residues in mouse (*Mm*) and budding yeast (*Sc*) subunits. b) The location of the variant amino acid residues (depicted space-filled in red) in EXOSC9 (depicted as ribbons in green) is indicated in the RNA exosome structure (PDB 6D6R) (Weick *et al.* 2018). c) Effect of EXOSC9 variants on ability of EXOSC9 to complement *rrp45Δ*. The EXOSC9 variants were expressed in an *rrp45Δ* yeast strain and subjected to plasmid shuffle experiments on media containing 5-FOA or control media. The growth of individual strains was compared with those that express control Rrp45 or wild-type EXOSC9 at the indicated days and temperatures. The steady-state protein levels of EXOSC9 variants were measured when expressed in the presence (d) or absence (e) of budding yeast Rrp45. For unknown reasons, the 48-KDa EXOSC9 results in an additional band with an apparent molecular weight of ∼75 KDa (hence its original name, PMSCL-75) in mammalian cells, which is replicated in yeast. The EXOSC9 protein levels (black arrowheads) for indicated strains were quantitated by quantitating both bands and plotted following normalization to Pgk1 (loading control; gray arrowhead) and compared with wild-type EXOSC2 level. The average of 2 biological replicates is plotted (bar) as well as the data points corresponding to the individual replicates (circles). *P*-values were determined by one-way ANOVAs.

Exome sequencing of patients with clinical characteristics of PCH1D identified a number of variants of the RNA exosome core subunit EXOSC9 (Fig. 5a and b) (Burns *et al.* 2018; Bizzari *et al.* 2020; Sakamoto *et al.* 2021). The characterization of these variants is limited to analyzing fibroblasts from a single patient homozygous for EXOSC9-L14P. The steady-state level of the EXOSC9 variant protein in these fibroblasts was lower than in control fibroblasts, and a few mRNAs were differentially expressed (Burns *et al.* 2018). Two other EXOSC9 variants that replace glycine 51 with arginine (EXOSC9-G51R) or leucine 80 with arginine (EXOSC9-L80R) have been identified in patients with PCH1D, but these variants have not been subjected to any functional analysis. The AlphaMissense pathogenicity prediction tool categorized all 3 variants as “likely pathogenic” (Supplementary Table 4).

To test whether these variants are damaging, we expressed EXOSC9 variants L14P, G51R, and L80R in an *rrp45Δ* yeast strain and performed a plasmid shuffle assay (Fig. 5c). The EXOSC9-L14P variant fails to complement the loss of yeast Rrp45 at both tested temperatures (30 and 37°C), indicating a severe functional defect in the RNA exosome. These data corroborate the previous observation that the EXOSC9-L14P variant causes defective RNA metabolism in the fibroblasts of an affected patient (Burns *et al.* 2018). Similarly, the EXOSC9-L80R variant shows a noticeable growth defect at 30°C and does not support growth at 37°C. In contrast, the EXOSC9-G51R variant supports growth much better than the other EXOSC9 variants examined and shows growth similar to control Rrp45 or wild-type EXOSC9.

The PCH-associated amino acid changes in EXOSC9 variants are predicted by DDMut and mCSM to destabilize the protein (Supplementary Table 4). Thus, we examined the EXOSC9 levels for wild-type and variant proteins when expressed in cells with yeast Rrp45 (Fig. 5d–f). The protein level of EXOSC9-L14P is reduced by ∼50% compared with wild-type EXOSC9. In contrast, a drastic reduction in protein levels to ∼10% of wild-type EXOSC9 is observed for both the EXOSC9-G51R and EXOSC9-L80R variants, suggesting that these amino acid changes may impair protein stability. We further tested the protein levels of EXOSC9-G51R and EXOSC9-L80R when expressed as the sole copy of EXOSC9. A similar reduction in protein levels for both variants was observed. Thus, our observations indicate that all 3 variants are damaging and affect either protein level or growth when expressed in yeast. Remarkably, there was no correlation between the effects on growth and protein level. The lethal EXOSC9-L14P protein variant was more abundant than the other 2 variants, and the protein levels of EXOSC9-L80R and EXOSC9-G51R were similar, but their effects on growth were very different. We suggest that L14 and L80 do not only contribute to protein stability but have direct roles in RNA exosome function. Consistent with this, EXOSC9 L14 is in close proximity to EXOSC3 and EXOSC9 L80 lies in the RNA conducting central channel.

## Discussion

The current study shows that 6 out of 9 core subunits of the yeast Exo9 complex can be replaced by mammalian (human or mouse) homologs. The replaceable subunits seem to cluster together on the one side of the complex, while the irreplaceable subunits cluster together on the other side of the complex (Fig. 1d and e). Several earlier studies have attempted to complement a yeast RNA exosome mutation with the human ortholog, but many of these studies were carried out with conditional alleles instead of complete deletions of the yeast genes. Our study is the first to systematically replace all Exo9 core subunits and to assess complementation with mouse subunits.

Our results generally align with previous observations that some human RNA exosome subunits can complement various yeast mutations. For example, the expression of human EXOSC1 and EXOSC2 was previously reported to complement *csl4-1* and *rrp4-1* conditional mutations, respectively (Mitchell *et al.* 1997; Baker *et al.* 1998); we extend this analysis to *csl4Δ* and *rrp4Δ*. Complementation of *rrp41* defects proved the most complicated and unpredictable. Previous work showed that human EXOSC4 can complement Rrp41-depleted yeast cells where Rrp41 is depleted using a repressible promoter (*gal::rrp41*) (Brouwer *et al.* 2001), and we replicated this result (Supplementary Fig. 1). However, the same EXOSC4 plasmid failed to complement *rrp41Δ*. We conclude that human EXOSC4 can substitute for some, but not all, aspects of Rrp41 function. Remarkably, myc-tagged mouse EXOSC4 complemented, but neither the untagged mouse EXOSC4 nor human EXOSC4 could complement. Notably, analysis of the protein levels of myc-MmEXOSC4 and MmEXOSC4 indicates that the level of untagged MmEXOSC4 is significantly reduced compared with myc-MmEXOSC4 (reduced to 17%; Supplementary Fig. 1), suggesting that the N-terminal myc tag may stabilize the MmEXOSC4 protein. Immunoblot analysis revealed equivalent signals for the complementing myc-MmEXOSC4 and the noncomplementing human EXOSC4 protein levels (Supplementary Fig. 1), suggesting that the myc tag may play more than just a stabilizing role in MmEXOSC4. We speculate that some other design for expressing EXOSC3, EXOSC5, and EXSCO8, such as a different epitope and/or C-terminal epitope tagging, could lead to complementation of the corresponding yeast deletions, but we cannot predict what design would be successful.

Our results also suggest that if complementation results in robust growth, several expression strategies give similar results (i.e. *csl4Δ*, *rrp4Δ*, and *rrp45Δ*). On the contrary, if complementation with a human ortholog fails, attempting the same complementation with another mammalian ortholog or with the addition of an epitope tag may be valuable. These results demonstrate that our ability to predict whether a mammalian gene can complement a yeast mutation is limited. For the 2 subunits (EXOSC4 and EXOC7) that only complemented with a subset of plasmid designs, we observed clear growth defect with all 3 variants tested, while for the robustly complementing subunits, some variants had little effect on growth. One possibility is that the EXOSC4- and EXOSC7-containing RNA exosomes are partly destabilized and any further damage is sensitively reflected in growth.

The variables that determine whether an RNA exosome subunit is replaceable remain unclear (Fig. 1d and e). For the replaceable subunits, which are depicted in color in Fig. 1d, the hybrid RNA exosome is expected to retain the critical protein–protein interactions between the neighboring RNA exosome subunits as well as several other proteins/cofactors required for RNA exosome function, including Rrp44/Dis3 (human DIS3 and DIS3L), Rrp6 (human EXOSC10), Mtr4 (human MTREX), Mpp6 (human MPHOSPH6), and Ski7 (human HBS1Lv3). These proteins primarily interact with either the cap subunits or, in the case of Rrp44, with the opposing “bottom” side of the ring. Among the 3 cap subunits, Csl4 and Rrp4 can be replaced by either of the tested mammalian counterparts (EXOSC1 and 2), suggesting that these cap subunits are able to maintain the intersubunit bridging contacts with the ring subunits, stabilize the complex, and accommodate interactions with the nuclear and cytoplasmic cofactors (Liu *et al.* 2006). On the bottom side, the replaceable part of the RNA exosome shares several contacts with the catalytic subunit Rrp44. This indicates that conserved interactions are retained in the hybrid exosome. Our findings revealing that irreplaceable subunits cluster together suggest that protein–protein interactions between neighboring subunits or with RNA exosome cofactors might have diverged between yeast and human. Thus, simultaneously replacing multiple irreplaceable subunits could be successful.

We utilized our humanized yeast model to show that most of the EXOSC variants associated with disease are damaging. Some of the damaging effects of the EXOSC variants resulted in reduced growth rate of the yeast cells, and some resulted in reduced protein level. For some of the EXOSC variants tested here, this is consistent with previous results. For other EXOSC variants, this is the first reported functional characterization. This analysis suggests that our humanized RNA exosome complements the previously used model system that involved making analogous mutations in the budding yeast subunits, in 3 aspects. First, due to low sequence similarities, some disease-linked variants are hard to analyze by making mutations in the orthologous budding yeast subunit genes. The EXOSC3-D132A variant has previously been modeled with *rrp40-S87A*. Similarly, the EXOSC9-L80R variant could potentially be modeled with *rrp45-T81R*. However, the D and L residues that are altered in the human variants are quite dissimilar to the S and T residues of the wild-type yeast proteins. In addition, some amino acids of EXOSC subunits do not readily align with the orthologous yeast protein. Our humanized model does not require identification of orthologous yeast residues and is thus applicable to all nonsynonymous EXOSC variants. Second, our observations indicate that the humanized yeast model provides more sensitive determinations of the disease-linked variants, and the damaging effects can be clearly distinguished. Several yeast RNA exosome variants have been reported to have only mild effects on growth, some of which require high temperatures or combination with another RNA exosome mutation to be readily apparent (Sterrett *et al.* 2021, 2023). In contrast, the corresponding variants are each clearly lethal in the humanized model. We speculate that the humanized yeast RNA exosome provides greater sensitivity to detect damaging variants because the RNA exosome is already destabilized by replacing 1 yeast subunit with the human homolog, which sensitizes the complex to further damage. Third, effects on protein levels are easily assayed in the humanized model using readily available commercial antibodies to detect the EXOSC subunits. In contrast, only a few anti-Rrp antibodies exist, and those that do exist are available in limited supply. Most of these advantages are maintained for complementation with MmEXOSC4, which differs from the human ortholog by only 20 amino acid residues. Thus, utilizing the humanized yeast model can enhance the genetic tools currently available for functional analysis of the RNA exosome variants and allow us to distinguish between pathogenic and benign variants more rapidly and easily.

We observed remarkably few correlations between effects on growth rate, protein steady-state levels, and the nature or severity of disease. Some EXOSC variants caused a reduction in steady-state protein levels and reduced growth rate, but other variants only affected protein levels with no detectable effect on growth and vice versa. Thus, while protein stability, reflected by a decrease in steady-state protein levels, may contribute to RNA exosome defects and exosomopathy symptoms, it is clearly not the only effect of many variants. Interestingly, some of the variants that cause severe growth defects but show relatively normal protein levels (e.g. EXOSC2-G30V, EXOSC2-R87Q, EXOSC9-L14P) affect residues that are in close proximity to neighboring RNA exosome subunits. Thus, these RNA exosome variants may impact protein–protein interactions between subunits. In support of this possibility, analysis of the orthologous yeast protein Rrp4-G58V showed reduced copurification with Rrp41 (Sterrett *et al.* 2021). In another case, the EXOSC9-L14P variant caused a decrease in the steady-state level of RNA exosome complexes in fibroblasts, suggesting defects in overall RNA exosome stability (Burns *et al.* 2018). A similar outcome is expected for the EXOSC2-R87Q variant. In addition, we observed no correlation between any of these effects and whether the variant is associated with PCH, SHRF, or CABAC, or with the severity of disease symptoms associated with the variant.

Along with the known defects caused by RNA exosome variants, our humanized yeast model examined the effect of previously uncharacterized variants (EXOSC2-R87Q, EXOSC2-R236Q, and EXOSC2-A143T) and novel candidate variants of unknown significance (EXOSC4-Y76N and EXOSC7-S229L). Overall, our humanized yeast model of exosomopathies provides a sensitive platform to help identify the damaging effects of patient-derived variants. This model can also be further utilized to explore the molecular mechanism(s) underlying functional defects in the RNA exosome.

## Supplementary Material

jkaf036_Supplementary_Data

## Data Availability

All data are included in the manuscript. Plasmids and yeast strains are available from the authors upon request. Supplemental material available at G3 online.
