# Supplementary material for: Humanized Saccharomyces cerevisiae provides a facile and effective tool to identify damaging human variants that cause exosomopathies
Source: G3 (Bethesda). 2025 Feb 21;15(4):jkaf036. doi: 10.1093/g3journal/jkaf036 (PMC12005145; doi:10.1093/g3journal/jkaf036)

Supplementary:

Table S1: List of plasmids used in this study.

Plasmid name	Plasmid description	Source / construction
pRS315	Empty vector with *LEU2* gene	(Sikorski and Hieter 1989)
pRS415	Empty vector with *LEU2* gene	(Sikorski and Hieter 1989)
pRS416	Empty vector with *URA3* gene	(Sikorski and Hieter 1989)
p415TEF	Empty vector with *LEU2* gene, *TEF1* promoter, and *CYC1* terminator	(Mumberg et al. 1995)
p425GPD	Empty vector with *LEU2* gene and *GPD* promoter and *CYC1* terminator	(Mumberg et al. 1995)
pRS315-TEF	Empty vector with *LEU2* gene, *TEF1* promoter, and *CYC1* terminator	*TEF1* promoter and *CYC1* terminator cloned into pRS315 using NEBuilder HiFi DNA Assembly kit (NEB)
pRS315-TEF-Myc	Empty vector with *LEU2* gene, *TEF1* promoter, 1x*Myc* tag, and *CYC1* terminator	*TEF1* promoter, 1x*Myc* tag, and *CYC1* terminator cloned into pRS315 using NEBuilder HiFi DNA Assembly kit (NEB)
pAv314	p425GPD-EXOSC1	(Schaeffer et al., 2009)
pAv1851	p415TEF-EXOSC2	Human EXOSC2 ORF obtained from GenScript and cloned in p415TEF digested with BamHI and XhoI.
pAv1852	p415TEF-EXOSC3	Human EXOSC3 ORF obtained from GenScript and cloned in p415TEF digested with BamHI and XhoI.
pAv1853	p415TEF-EXOSC4	Human EXOSC4 ORF obtained from GenScript and cloned in p415TEF digested with BamHI and XhoI.
pAv1854	p415TEF-EXOSC5	Human EXOSC5 ORF obtained from GenScript and cloned in p415TEF digested with BamHI and XhoI.
pAv1855	p415TEF-EXOSC6	Human EXOSC6 ORF obtained from GenScript and cloned in p415TEF digested with BamHI and XhoI.
pAv1856	p415TEF-EXOSC7	Human EXOSC7 ORF obtained from GenScript and cloned in p415TEF digested with BamHI and XhoI.
pAv1857	p415TEF-EXOSC8	Human EXOSC8 ORF obtained from GenScript and cloned in p415TEF digested with BamHI and XhoI.
pAv1858	p415TEF-EXOSC9	Human EXOSC9 ORF obtained from GenScript and cloned in p415TEF digested with BamHI and XhoI.
pAC4431	pRS315-TEF-MmEXOSC1	Mouse EXOSC1 ORF amplified from murine N2a cDNA and cloned into pRS315-TEF using NEBuilder HiFi DNA Assembly kit (NEB)

pAC4432	pRS315-TEF-MmEXOSC2	Mouse EXOSC2 ORF amplified from murine N2a cDNA and cloned into pRS315-TEF using NEBuilder HiFi DNA Assembly kit (NEB)
pAC4433	pRS315-TEF-MmEXOSC3	Mouse EXOSC3 ORF amplified from murine N2a cDNA and cloned into pRS315-TEF using NEBuilder HiFi DNA Assembly kit (NEB)
pAC4434	pRS315-TEF-MmEXOSC4	Mouse EXOSC4 ORF amplified from murine N2a cDNA and cloned into pRS315-TEF using NEBuilder HiFi DNA Assembly kit (NEB)
pAC4435	pRS315-TEF-MmEXOSC5	Mouse EXOSC5 ORF amplified from murine N2a cDNA and cloned into pRS315-TEF using NEBuilder HiFi DNA Assembly kit (NEB)
pAC4436	pRS315-TEF-MmEXOSC6	Mouse EXOSC6 ORF amplified from murine N2a cDNA and cloned into pRS315-TEF using NEBuilder HiFi DNA Assembly kit (NEB)
pAC4437	pRS315-TEF-MmEXOSC7	Mouse EXOSC7 ORF amplified from murine N2a cDNA and cloned into pRS315-TEF using NEBuilder HiFi DNA Assembly kit (NEB)
pAC4438	pRS315-TEF-MmEXOSC8	Mouse EXOSC8 ORF amplified from murine N2a cDNA and cloned into pRS315-TEF using NEBuilder HiFi DNA Assembly kit (NEB)
pAC4439	pRS315-TEF-MmEXOSC9	Mouse EXOSC9 ORF amplified from murine N2a cDNA and cloned into pRS315-TEF using NEBuilder HiFi DNA Assembly kit (NEB)
pAC4449	pRS315-TEF-Myc-MmEXOSC1	Mouse EXOSC1 ORF amplified from murine N2a cDNA and cloned into pRS315-TEF-Myc using NEBuilder HiFi DNA Assembly kit (NEB)
pAC4450	pRS315-TEF-Myc-MmEXOSC2	Mouse EXOSC2 ORF amplified from murine N2a cDNA and cloned into pRS315-TEF-Myc using NEBuilder HiFi DNA Assembly kit (NEB)
pAC4451	pRS315-TEF-Myc-MmEXOSC3	Mouse EXOSC3 ORF amplified from murine N2a cDNA and cloned into pRS315-TEF-Myc using NEBuilder HiFi DNA Assembly kit (NEB)
pAC4452	pRS315-TEF-Myc-MmEXOSC4	Mouse EXOSC4 ORF amplified from murine N2a cDNA and cloned into pRS315-TEF-Myc using NEBuilder HiFi DNA Assembly kit (NEB)
pAC4453	pRS315-TEF-Myc-MmEXOSC5	Mouse EXOSC5 ORF amplified from murine N2a cDNA and cloned into pRS315-TEF-Myc using NEBuilder HiFi DNA Assembly kit (NEB)
pAC4454	pRS315-TEF-Myc-MmEXOSC6	Mouse EXOSC6 ORF amplified from murine N2a cDNA and cloned into pRS315-TEF-Myc using NEBuilder HiFi DNA Assembly kit (NEB)
pAC4455	pRS315-TEF-Myc-MmEXOSC7	Mouse EXOSC7 ORF amplified from murine N2a cDNA and cloned into pRS315-TEF-Myc using NEBuilder HiFi DNA Assembly kit (NEB)
pAC4456	pRS315-TEF-Myc-MmEXOSC8	Mouse EXOSC8 ORF amplified from murine N2a cDNA and cloned into pRS315-TEF-Myc using NEBuilder HiFi DNA Assembly kit (NEB)

pAC4457	pRS315-TEF-Myc-MmEXOSC9	Mouse EXOSC9 ORF amplified from murine N2a cDNA and cloned into pRS315-TEF-Myc using NEBuilder HiFi DNA Assembly kit (NEB)
pAv2040	p415TEF-EXOSC2-G30V	G30V mutation was introduced in p415TEF-EXOSC2 (pAv1851) using QuikChange Site-Directed Mutagenesis Kit (Agilent).
pAv2043	p415TEF-EXOSC2-R87Q	R87Q mutation was introduced in p415TEF-EXOSC2 (pAv1851) using QuikChange Site-Directed Mutagenesis Kit (Agilent).
pAv2042	p415TEF-EXOSC2-A143T	A143T mutation was introduced in p415TEF-EXOSC2 (pAv1851) using QuikChange Site-Directed Mutagenesis Kit (Agilent).
pAv2041	p415TEF-EXOSC2-G198D	G198D mutation was introduced in p415TEF-EXOSC2 (pAv1851) using QuikChange Site-Directed Mutagenesis Kit (Agilent).
pAv2044	p415TEF-EXOSC2-R236Q	R236Q mutation was introduced in p415TEF-EXOSC2 (pAv1851) using QuikChange Site-Directed Mutagenesis Kit (Agilent).
pAC4464	pRS315-TEF-Myc-MmEXOSC4-Y76N	Y76N mutation was introduced in pRS315-TEF-Myc-MmEXOSC4 (pAC4452) using QuickChange Site-Directed Mutagenesis Kit (Agilent)
pAC4465	pRS315-TEF-Myc-MmEXOSC4-L187P	L187P mutation was introduced in pRS315-TEF-Myc-MmEXOSC4 (pAC4452) using QuickChange Site-Directed Mutagenesis Kit (Agilent)
pAv2048	p415TEF-EXOSC7-S229L	S229L mutation was introduced in p415TEF-EXOSC7 (pAv1856) using QuikChange Site-Directed Mutagenesis Kit (Agilent).
pAv2049	p415TEF-EXOSC9-L14P	L14P mutation was introduced in p415TEF-EXOSC9 (pAv1858) using QuikChange Site-Directed Mutagenesis Kit (Agilent).
pAv2050	p415TEF-EXOSC9-G51R	G51R mutation was introduced in p415TEF-EXOSC9 (pAv1858) using QuikChange Site-Directed Mutagenesis Kit (Agilent).
pAv2051	p415TEF-EXOSC9-L80R	L80R mutation was introduced in p415TEF-EXOSC9 (pAv1858) using QuikChange Site-Directed Mutagenesis Kit (Agilent).
pAC2981	pRS315-CSL4	CSL4 gene (promoter, ORF, 3'UTR) amplified from W303 gDNA, digested with BamHI and SacI and cloned into pRS315 cut with same enzymes
pAC3656	pRS315-RRP4	Sterrett et al. RNA (2021)
pAC3652	pRS315-RRP40	Sterrett et al. RNA (2021)
pAC4179	pRS315-RRP41	Fasken et al. JBC (2024)
pAC3482	pRS315-RRP46	Slavotinek et al. Hum. Mol. Genet. (2020)
pAC2985	pRS315-MTR3	*MTR3* gene (promoter, ORF, 3'UTR) amplified from W303 gDNA, digested with BamHI and SacI and cloned into pRS315 cut with same enzymes

pAC2953	pRS315-*RRP42*	*RRP42* gene (promoter, ORF, 3'UTR) amplified from W303 gDNA, digested with BamHI and SacI and cloned into pRS315 cut with same enzymes
pAC2965	pRS315-*RRP43*	*RRP43* gene (promoter, ORF, 3'UTR) amplified from W303 gDNA, digested with BamHI and SacI and cloned into pRS315 cut with same enzymes
pAC2977	pRS315-*RRP45*	*RRP45* gene (promoter, ORF, 3'UTR) amplified from W303 gDNA, digested with BamHI and SacI and cloned into pRS315 cut with same enzymes

Table S2: List of oligonucleotides used in this study.

Oligo	Sequence
EXOSC2 Forward	agcaatctaatctaagttttGGATCCATAACCATGGCTATG
EXOSC2 Reverse	tatcgataagcttgatatcgaattcCTCGAGTCACCCTTCTTG
EXOSC3 Forward	agcaatctaatctaagttttGGATCCATAACCATGGCTG
EXOSC3 Reverse	tatcgataagcttgatatcgaattcCTCGAGTCAGGATTCAGC
EXOSC4 Forward	agcaatctaatctaagttttGGATCCATAACCATGGCTG
EXOSC4 Reverse	tatcgataagcttgatatcgaattcCTCGAGTCAATCACCCAAC
EXOSC5 Forward	agcaatctaatctaagttttGGATCCATAACCATGGAAG
EXOSC5 Reverse	tatcgataagcttgatatcgaattcCTCGAGTCAGGATTTGGAG
EXOSC6 Forward	agcaatctaatctaagttttGGATCCATAACCATGCCAG
EXOSC6 Reverse	tatcgataagcttgatatcgaattcCTCGAGTCATGGTTGGGC
EXOSC7 Forward	agcaatctaatctaagttttGGATCCATAACCATGGCTAG
EXOSC7 Reverse	tatcgataagcttgatatcgaattcCTCGAGTCAGCCTAAGAAAC
EXOSC8 Forward	agcaatctaatctaagttttGGATCCATAACCATGGCC
EXOSC8 Reverse	tatcgataagcttgatatcgaattcCTCGAGTCACTTTGGCTTC
EXOSC9 Forward	agcaatctaatctaagttttGGATCCATAACCATGAAGGAAAC
EXOSC9 Reverse	tatcgataagcttgatatcgaattcCTCGAGTCAATTGGCGGC
EXOSC2 G30V Forward	cagtggtgatagtgtcgactggaacaaccaagtgc
EXOSC2 G30V Reverse	gcacttggttgttccagtcgacactatcaccactg
EXOSC2 G198D Forward	cagatgaaaccgttgttatccaagatgactgaggcac
EXOSC2 G198D Reverse	gtgcctcagtcatcttggataacaacggtttcatctg
EXOSC2 A143T Forward	agaaaacagcttggacttcagtagaaatcaaatcaccttcttg
EXOSC2 A143T Reverse	caagaaggtgatttgatttctactgaagtccaagctgttttct
EXOSC2 R87Q Forward	tttgttggacttcagtgatctgaccgacgacaatgtcacc
EXOSC2 R87Q Reverse	ggtgacattgtcgtcggtcagatcactgaagtccaacaaa
EXOSC2 R236Q Forward	accaaagagatgatacagtttctcaactgagagataacttctctgtcagccaaag
EXOSC2 R236Q Reverse	ctttggctgacagagaagttatctctcagttgagaaactgtatcatctctttggt
MmEXOSC4 L187P Forward	aagctggcaacaaggctggggccaattgtggaccac
MmEXOSC4 L187P Reverse	gtggtccacaattggccccagccttgttgccagctt
MmEXOSC4 Y76N Forward	gaaggtggctgaactgttctgacagttcactagag
MmEXOSC4 Y76N Reverse	ctctagtgaactgtcagaacagttcagccaccttc
EXOSC7 S229L Forward	caacacccttggaggtaactaagaccaacaaagaagccaa

EXOSC7 Y76N Reverse	ttggcttctttgttggtcttagttacctccaagggtgttg
EXOSC9 L14P Forward	ctcttcaatggccctcaacgggaatcttctttcacagtttgacaa
EXOSC9 L14P Reverse	ttgtcaaactgtgaaagaagattcccgttgagggccattgaagag
EXOSC9 G51R Forward	caagactctggtcttacgtaattcaacaatacaacacccat
EXOSC9 G51R Reverse	atgggtgttgtattgttgaattacgtaagaccagagtcttg
EXOSC9 L80R Forward	gagcagccatttgtgacaattccctgttgaaaaacaaaataccttcgg
EXOSC9 L80R Reverse	ccgaaggtattttgtttttcaacagggaattgtcacaaatggctgctc

Table S3: List of yeast strains used in this study.

name	genotype
yAv1047	*matA, ura3-Δ0, leu2-Δ0, his3-Δ1, lys2-Δ0, csl4Δ::kanMX [CSL4, URA3]*
yAv1103	*matα, ura3-Δ0, leu2-Δ0, his3-Δ1, lys2-Δ0, rrp4Δ::kanMX [RRP4, URA3]*
yAv1107	*matA, ura3-Δ0, leu2-Δ0, his3-Δ1, lys2-Δ0, rrp40Δ::kanMX [RRP40, URA3]*
yAv2493	*matα, ura3-Δ0, leu2-Δ0, his3-Δ1, rrp41Δ::kanMX [RRP41, URA3]*
yAv4920	*matA, ura3-Δ0, leu2-Δ0, his3-Δ1, rrp46Δ::kanMX [RRP46, URA3]*
yAv4443	*matA, ura3-Δ0, leu2-Δ0, his3-Δ1, lys2-Δ0, mtr3Δ::kanMX [MTR3, URA3]*
yAv2506	*matα, ura3-Δ0, leu2-Δ0, his3-Δ1, rrp42Δ::kanMX [RRP42, URA3]*
yAv2071	*matA, ura3-Δ0, leu2-Δ0, his3-Δ1, rrp43Δ::kanMX [RRP43, URA3]*
yAv1410	*matα, ura3-Δ0, leu2-Δ0, his3-Δ1, rrp45Δ::kanMX [RRP45, URA3]*
ACY2880	*matA, ura3-Δ0, leu2-Δ0, his3-Δ1, met15-Δ0, csl4Δ::kanMX [CSL4, URA3]*
ACY2856	*matA, ura3-Δ0, leu2-Δ0, his3-Δ1, met15-Δ0, rrp4Δ::kanMX [RRP4, URA3]*
ACY2859	*matA, ura3-Δ0, leu2-Δ0, his3-Δ1, met15-Δ0, rrp40Δ::kanMX [RRP40, URA3]*
ACY2862	*matA, ura3-Δ0, leu2-Δ0, his3-Δ1, met15-Δ0, rrp41Δ::kanMX [RRP41, URA3]*
ACY2874	*matA, ura3-Δ0, leu2-Δ0, his3-Δ1, met15-Δ0, rrp46Δ::kanMX [RRP46, URA3]*
ACY2877	*matA, ura3-Δ0, leu2-Δ0, his3-Δ1, met15-Δ0, mtr3Δ::kanMX [MTR3, URA3]*
ACY2865	*matA, ura3-Δ0, leu2-Δ0, his3-Δ1, met15-Δ0, rrp42Δ::kanMX [RRP42, URA3]*
ACY2869	*matA, ura3-Δ0, leu2-Δ0, his3-Δ1, met15-Δ0, rrp43Δ::kanMX [RRP43, URA3]*
ACY2871	*matA, ura3-Δ0, leu2-Δ0, his3-Δ1, met15-Δ0, rrp45Δ::kanMX [RRP45, URA3]*

Table S4: Prediction of pathogenicity and protein stability of the RNA exosome variants

RNA exosome variants	AlphaMissense prediction (Score)	PolyPhen-2 prediction	DDMut variant stability (ΔΔG) kcal/mol	mCSM variant stability (ΔΔG) kcal/mol
EXOSC2-G30V	Likely pathogenic 0.996	Probably damaging	Destabilizing -2.22	Destabilizing -0.63
EXOSC2-G198D	Likely pathogenic 0.997	Probably damaging	Destabilizing -2.28	Highly destabilizing -2.504
EXOSC2-A143T	Likely pathogenic 0.744	Probably damaging	Destabilizing -1.45	Destabilizing -1.583
EXOSC2-R87Q	Ambiguous 0.449	Probably damaging	Destabilizing -0.81	Destabilizing -1.134
EXOSC2-R236Q	Likely pathogenic 0.7	Probably damaging	Destabilizing -0.86	Destabilizing -1.463
EXOSC4-L187P	Likely pathogenic 0.941	Probably damaging	Destabilizing -2.86	Destabilizing -1.801
EXOSC4-Y76N	Likely pathogenic 0.99	Probably damaging	Destabilizing -3.08	Destabilizing -1.571
EXOSC7-S229L	Ambiguous 0.469	Benign	Stabilizing 1.05	Stabilizing 0.019
EXOSC9-L14P	Likely pathogenic 0.987	Probably damaging	Destabilizing -3.2	Destabilizing -1.056
EXOSCP-G51R	Likely pathogenic 0.978	Probably damaging	Destabilizing -0.0	Destabilizing -0.935
EXOSC9-L80R	Likely pathogenic 0.958	Probably damaging	Destabilizing -1.78	Destabilizing -1.688

Figure S1: Complementation of *rrp41* strains. A. Expression of human EXOSC4 can complement a *gal::rrp41* strain, confirming published results. Two variants cause a reduction in growth, confirming results with the mouse variants in Figure 3. This figure uses strain p118 from Mitchell *et al.* 1997 Cell 91:457–466. **B.** Western blot comparing the expression levels of tagged and untagged mouse EXOSC4. Human EXOSC4 is included as a control. Human and mouse EXOSC4 differ in only 10 of their 245 residues. The western blot is probed with a polyclonal antibody raised against the first 50 residues of human EXOSC4. In this region, mouse EXOSC4 differs by one amino acid residue (V14 in human and I14 in mouse). While human EXOSC4 gives a stronger signal than mouse EXOSC4, we did not directly compare the human and mouse protein levels because of this one residue difference.

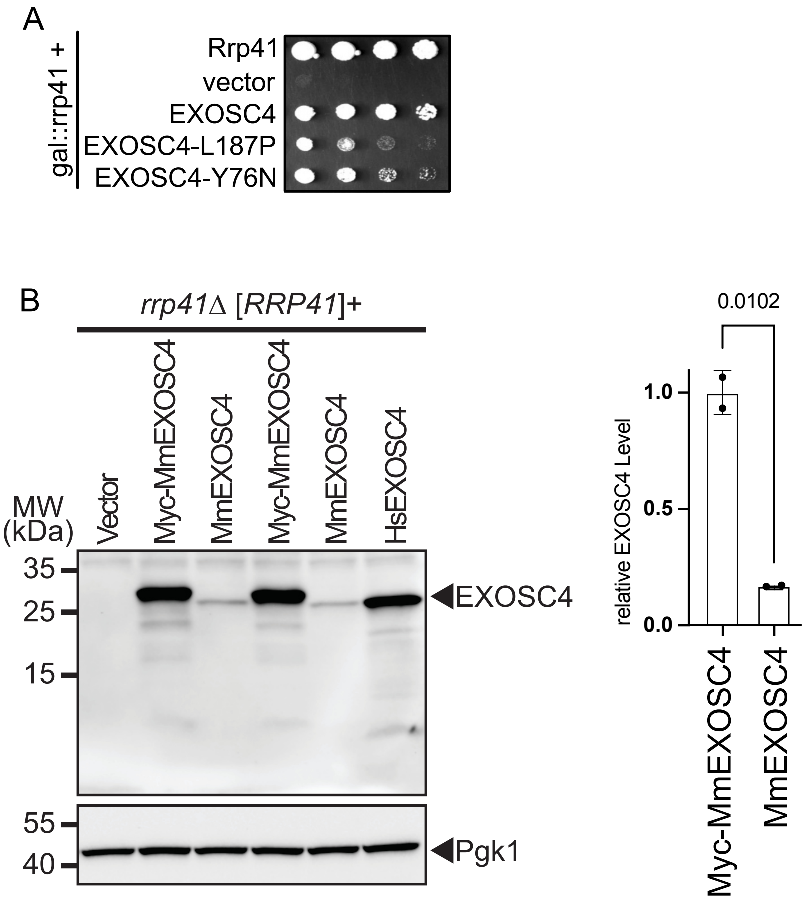

Figure S2: The RNA exosome is highly conserved between yeast and animals. Shown at the top is a superposition of published human (6D6R in orange) and yeast (6FSZ in aqua blue) RNA exosome core structures. The graph shows sequence similarities between the human RNA exosome and the orthologs from yeast (aqua blue) and mouse (grey bars).

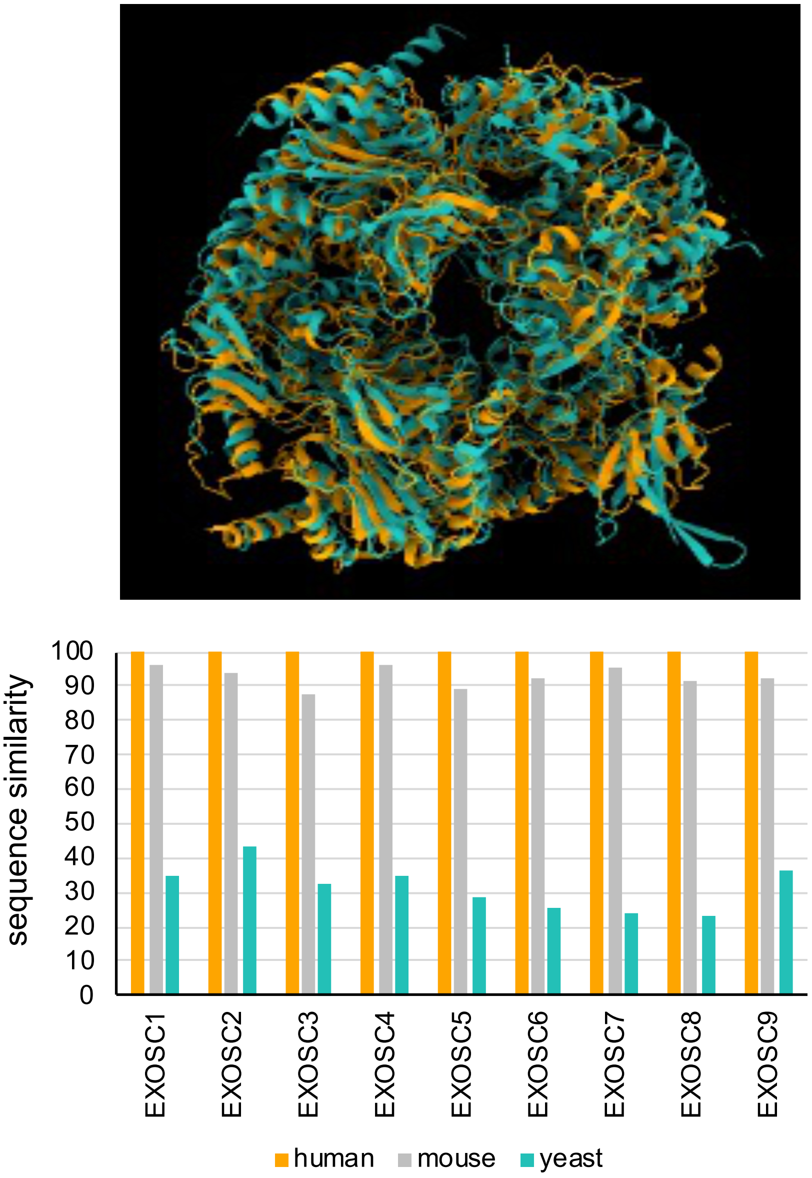

Figure S3: Structural representations and locations of the replaceable RNA exosome subunits. (A). Csl4 Rrp4 and Rrp45 can be replaced by both the human and mouse subunits and this complementation results in growth that is similar to wild type. (B) Rrp41, Rrp43, and Mtr3 can be replaced by either the human ortholog or the myc-tagged mouse ortholog and this compelmentation results in slow growth. Colors are as in figure 1 D and E.

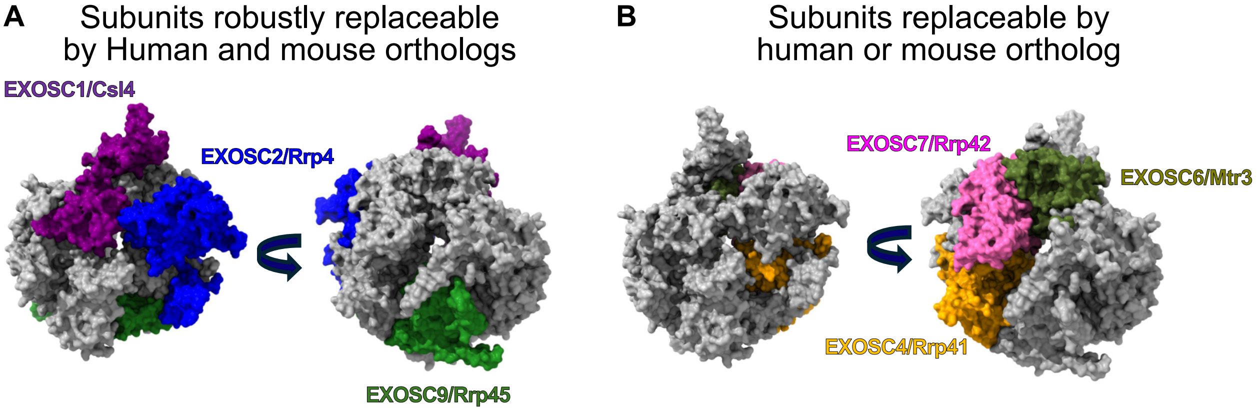

Supplement: jkaf036_Supplementary_Data [file jkaf036_supplementary_data.pdf]
